# AMSEANet: An Edge-Guided Adaptive Multi-Scale Network for Image Splicing Detection and Localization

**DOI:** 10.3390/s25206494

**Published:** 2025-10-21

**Authors:** Yuankun Yang, Yueshun He, Xiaohui Ma, Wei Lv, Jie Chen, Hongling Wang

**Affiliations:** 1 School of Artificial Intelligence and Information Engineering, East China University of Technology, Nanchang 330013, China; 2023110197@ecut.edu.cn (Y.Y.); 2023110187@ecut.edu.cn (X.M.); 2023110202@ecut.edu.cn (W.L.); 2 School of Surveying and Geoinformation Engineering, East China University of Technology, Nanchang 330013, China; codercjie@ecut.edu.cn; 3 Jiangxi Engineering Laboratory on Radioactive Geoscience and Big Data Technology, School of Software, East China University of Technology, Nanchang 330013, China; wanghl@ecut.edu.cn

**Keywords:** image splicing localization, edge-awareness, cross-scale dense fusion, synergistic enhancement network

## Abstract

In image splicing tamper detection, forgery operations simultaneously introduce macroscopic semantic inconsistencies and microscopic tampering artifacts. Conventional methods often treat semantic understanding and low-level artifact perception as separate tasks, which impedes their effective synergy. Meanwhile, frequency-domain information, a crucial clue for identifying traces of tampering, is frequently overlooked. However, a simplistic fusion of frequency-domain and spatial features can lead to feature conflicts and information redundancy. To resolve these challenges, this paper proposes an Adaptive Multi-Scale Edge-Aware Network (AMSEANet). This network employs a synergistic enhancement cascade architecture, recasting semantic understanding and artifact perception as a single, frequency-aware process guided by deep semantics. It leverages data-driven adaptive filters to precisely isolate and focus on edge artifacts that signify tampering. Concurrently, the dense fusion and enhancement of cross-scale features effectively preserve minute tampering clues and boundary details. Extensive experiments demonstrate that our proposed method achieves superior performance on several public datasets and exhibits excellent robustness against common attacks, such as noise and JPEG compression.

## 1. Introduction

**Figure 1:**
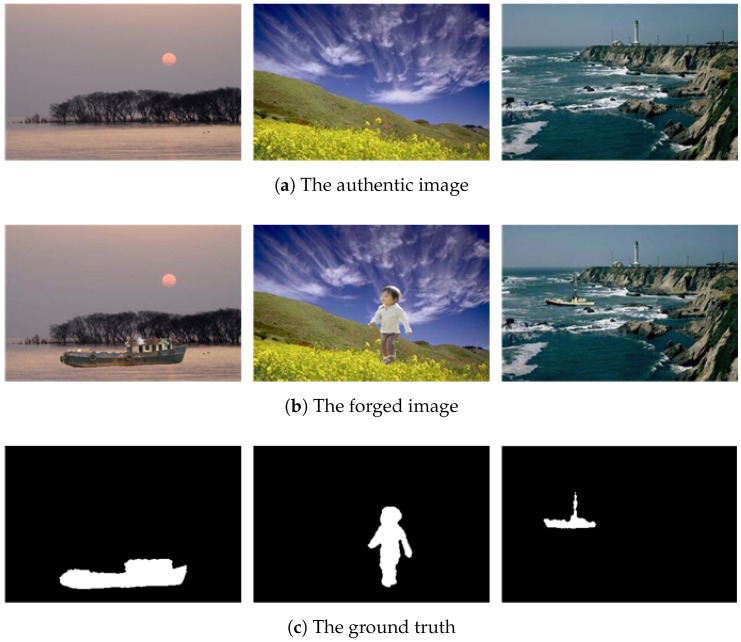
Example of image splicing forgery. (**a**) The authentic image. (**b**) The forged image. (**c**) The ground truth.

With the rapid development of digital image processing technologies, current techniques for digital image generation, editing, and dissemination have permeated every aspect of modern life. As the primary medium for information transmission, images play an indispensable role in critical domains such as photojournalism, judicial forensics, and national security, where their authenticity and integrity directly impact public perception and social stability. However, the evolution of image editing software, typified by Adobe Photoshop, has significantly lowered the barrier to creating sophisticated forgeries. This enables even non-expert users to easily produce highly realistic image manipulations. Among these techniques, image splicing—which involves constructing false semantic content by copying and pasting regions from multiple source images—has become a primary method of manipulation. It is important to note that while image editing serves many legitimate creative purposes, the scope of this paper is focused on the forensic challenge of detecting “malicious forgeries” intended to deceive, as illustrated in Figure 1. Such tampering not only disrupts social order but can also be exploited for criminal activities, including political manipulation and financial fraud, thereby highlighting the urgent need for robust image splicing detection technologies.

Compared to other tampering methods, such as copy-move or content removal, image splicing achieves semantic deception by fusing content from different image domains, which significantly increases its detection difficulty. On the one hand, forgers can employ sophisticated post-processing techniques (e.g., noise addition, color adjustment) to eliminate the traces of physical inconsistency relied upon by traditional detection methods. On the other hand, the complex semantic relationships and cross-domain feature discrepancies among source images often cause existing algorithms to generate false positives and false negatives when localizing splicing boundaries and tracing the provenance of forged regions. Furthermore, deep learning-driven generative techniques, such as GAN-based image synthesis, further blur the visual boundaries between authentic and forged content, placing higher demands on the robustness and generalization capabilities of detection models.

During their formation, digital images acquire unique digital imprints from the optical characteristics of the imaging device [1], the physical environment, and digital processing procedures [2]. These medium-specific features, unrelated to the visual content, are termed “image digital fingerprints” due to their uniqueness and can serve as identifiers for image source tracing. In the field of digital image forensics, the core principle of splicing forgery detection lies in identifying attribute inconsistencies between the tampered and authentic regions. This inconsistency arises from the inherent feature differences between various source images. Existing research has developed multiple feature extraction techniques to capture such discrepancies, which are mainly categorized into two approaches: traditional handcrafted feature methods [3,4], and deep learning-based CNN feature extraction methods [5,6,7,8]. Traditional methods involve extracting features through manual design, followed by comparative or statistical analysis of these features to determine if an image has been tampered with and to locate the spliced region. However, the performance of these traditional image forensic methods heavily relies on predefined, singular image fingerprints (such as sensor noise or traces of interpolation). These features are vulnerable to common post-processing operations like blurring and JPEG compression, which can lead to feature invalidation and result in missed detections.

Inspired by the success of deep learning in other computer vision domains, researchers began to actively explore its application in image tamper detection [9]. The essence of image tamper detection lies in the synergy of localizing tampering artifacts (e.g., inconsistent edge seams, abnormal noise patterns) and verifying the consistency of pixel origins. This task requirement spurred the development of patch-level analysis frameworks [10,11]. These methods segment the image into independent small patches for processing, relying on local feature extraction mechanisms to avoid excessive focus on semantic content unrelated to traces of tampering, thereby enhancing sensitivity to subtle artifacts like resampling patterns and compression noise [12]. However, their core deficiency lies in a fragmented processing logic. While the local field of view of a single patch can isolate irrelevant semantic content, it consequently fails to capture tamper-related spatial information and global semantic associations (such as cross-regional physical plausibility and lighting consistency), thus ignoring the relationships between patches. For instance, artifacts are prone to appear at patch boundaries, necessitating post-processing algorithms for repair; simultaneously, the patch size must be manually preset, making it difficult to adapt to multi-scale tampering scenarios (e.g., small targets being missed or large targets being fragmented). More critically, these methods rely on a disjointed “divide → classify → merge” workflow, which decouples the feature extraction and localization tasks, thereby weakening the model’s generalization ability for complex tampering patterns (such as low-contrast splicing).

To achieve better global modeling, researchers proposed methods based on end-to-end networks [13,14,15,16,17]. Instead of partitioning the image, they treat splicing tamper detection as a pixel-wise binary classification problem: classifying each pixel as either belonging to a “tampered region” or an “authentic region”. Such methods can accept images of any size as input, thereby eliminating the feature discontinuities caused by manual patching and overcoming the drawbacks of patch-based approaches to some extent. However, constrained by the inherent architecture of CNNs, deep networks still prioritize local features and have insufficient global semantic verification capabilities. Concurrently, local and global information are difficult to effectively complement, making it challenging to capture the contextual associations of traces of tampering at different scales.

Against this backdrop, dual-branch network architectures emerged [18,19,20,21], attempting to break the limitations of single-stream modeling through heterogeneous feature fusion strategies. These methods typically construct parallel processing paths to separately extract spatial-domain features (e.g., RGB image features) and frequency-domain features (e.g., noise, compression artifacts), compensating for the global perception shortcomings of CNNs through cross-modal feature interaction. However, their core bottleneck is the information isolation introduced by the parallel branches. In this paradigm, the frequency-domain analysis branch operates without knowledge of the image’s semantic content, while the spatial-domain branch cannot leverage low-level artifact clues to aid its judgment. This ’working in isolation’ during the feature extraction stage poses a significant challenge for subsequent fusion modules (whether simple concatenation or complex attention mechanisms), making it difficult to fundamentally resolve the feature conflicts and semantic disconnects caused by information isolation. Furthermore, existing attention mechanisms struggle to establish a synergistic enhancement relationship between local details (such as edge artifacts) and global semantics (such as the distribution of the tampered region), leading to frequent issues of missed small-scale tampered targets and blurred boundary localization.

To address the aforementioned shortcomings, this paper proposes an Adaptive Multi-Scale Edge-Aware Network (AMSEANet). This network discards the inherent drawbacks of the traditional parallel dual-branch paradigm in feature interaction and innovatively designs a single-backbone synergistic enhancement framework aimed at organically unifying the two previously separate tasks of deep semantic understanding and low-level tampering artifact perception. A fundamental prerequisite for realizing this synergistic framework is to address the problem of information dilution, where key features are inherently diluted as they pass through deep layers of a single-backbone network. To tackle this challenge, we first introduce the Cross-Scale Dense Residual Fusion Block (CSDRFBlock). This module forms the foundation for feature preservation, ensuring the efficient cross-level transmission of critical clues, such as minute tampering artifacts, from the source. Building upon this high-fidelity feature stream, the network must then precisely discern traces of tampering from a complex background. Considering the limitations of traditional fixed filters for this task, we subsequently designed the Edge-Aware Spatial-Frequency Fusion Module (ESFFM), driven by an Adaptive Smooth Filter. This module takes the enhanced features from the preceding module as input and dynamically focuses on authentic tampering boundaries in a data-driven manner. Finally, to effectively integrate and reorganize these multi-stage enhanced and refined features in the decoder, we propose a novel cross-scale attention mechanism (MGFA). This mechanism facilitates the seamless fusion of features from different scales and semantic levels, thereby ensuring the global consistency and boundary precision of the final segmentation mask.

In summary, the main contributions of this paper are as follows:

1. We propose a novel model architecture for image splicing detection, built upon a “spatial-guided frequency perception” philosophy and implemented within a single-backbone synergistic framework. This architecture fundamentally differs from traditional dual-branch networks, which suffer from isolated feature extraction and late-stage fusion. Instead, our framework uses deep spatial semantics to actively guide the perception and enhancement of high-frequency artifacts within a unified feature stream, which is realized through a combination of supervision with an edge loss, adaptive frequency perception, and multi-scale feature fusion.
2. We design a CSDRFBlock that employs a progressive cascade mechanism to drive the dynamic inheritance and reinforcement of multi-scale features. This avoids the feature conflicts typical of dual-branch fusion and significantly improves the cross-level transmission efficiency of critical clues, such as small-scale tampering artifacts.
3. We design an MGFA mechanism. Through strategies including bi-directional channel-spatial feature calibration, local-global semantic focusing, and dynamic weight aggregation, it establishes strong relationships between tampered regions and the background within multi-scale feature maps, effectively mitigating issues of blurred boundaries and semantic disconnection.
4. We design an ESFFM with an ASF at its core. This module learns to optimally separate high- and low-frequency tampering artifacts in a data-driven manner, overcoming the limitations of traditional fixed filters and significantly improving the model’s localization precision for tampering boundaries.

## 2. Related Work

The core strategy of traditional methods focuses on the extraction and analysis of specific image fingerprints, which are mainly divided into three technical approaches. The first is based on sensor pattern noise (SPN) [1,2], which performs source detection by analyzing the inherent noise characteristics of imaging devices; however, a convergence of noise features among similar devices can lead to detection failure. The second is based on the quantization effects of Discrete Cosine Transform (DCT) coefficients. Zhao et al. (2014) [4] proposed an image splicing forgery detection method based on a two-dimensional Markov model, which is applied in the block DCT domain and the Discrete Multiwavelet Transform (DMWT) domain, and utilizes cross-domain characteristics as classification features. Although this method offers enhanced robustness against median filtering and JPEG compression, it is inherently complex. The third category of methods focuses on detecting traces introduced by geometric transformations. For example, Popescu and Farid [22] proposed a method to detect image resampling (such as scaling and rotation). This technique identifies tampered regions by analyzing the periodic correlations among pixels. Since these traditional methods primarily target a single type of tampering feature and rely on the design of handcrafted statistical features, they can be easily circumvented by targeted manipulations, resulting in suboptimal detection performance.

In recent years, deep learning methods have been implemented to solve numerous scientific problems and have gained immense popularity. This is because their performance in classification problems, regression problems, and segmentation challenges has proven to be outstanding. Consequently, many deep learning-based methods for image tamper detection have been proposed. Initially, researchers focused on using convolutional neural networks for splicing tamper localization at the patch level. Bappy et al. [10] proposed a hybrid architecture combining a CNN encoder with an LSTM to achieve tampered region localization by simultaneously extracting spatial and frequency-domain features. Experiments showed that it could achieve pixel-level tamper localization without relying on handcrafted feature design, surpassing traditional handcrafted feature methods in detection accuracy. Xiao et al. [11] proposed a coarse-to-fine convolutional neural network (C2R-Net) based on non-overlapping image patches, which segments the input image into fixed-size, non-overlapping patches, uses a CNN to extract multi-scale features, and combines this with an adaptive clustering algorithm to localize tampered regions. However, such methods require segmenting the image into independent small patches for processing, leading to significant redundant computation, and the local field of view of a single patch makes it difficult to capture global semantic associations.

The proposal of Fully Convolutional Networks (FCNs) directly addressed the aforementioned bottlenecks. Through a fully convolutional encoder–decoder architecture, they achieve end-to-end mapping from an entire input image to a pixel-level output, completely abandoning the patching operation. Liu and Pun [15] proposed a splicing tamper localization model based on a cascade of a multi-scale Fully Convolutional Network and a Conditional Random Field (FCNs–CRF). It extracts deep features using three FCNs with different scale sensitivities and combines them with a CRF to adaptively fuse the multi-scale prediction results. However, FCNs–CRF lacks the ability to learn the tampered region’s edges, leading to suboptimal feature fusion performance. Considering that image splicing detection is essentially a pixel-level binary classification task, Bi [13] proposed a method combining a Ringed Residual U-Net (RRU-Net). RRU-Net constructs cross-level feature interaction channels through forward propagation in residual paths and backward connections in residual feedback paths, achieving multi-level reuse of features within the network. However, experimental results showed that on the COLUMBIA dataset, the detected tampered regions exhibited discontinuities and blurred boundaries, indicating a limited ability to capture fine-grained structures. Although the ManTra-Net model proposed by Wu et al. [14] extracts feature patterns of 385 known manipulation types through self-supervised learning, it lacks detailed information for localizing the tampered region.

In recent years, multi-scale strategies have been widely applied across various computer vision tasks. For instance, models such as DDMSNet [23] and GridFormer [24] adopt strategies that process input image pyramids, while others like ESTINet [25] construct feature pyramids internally through encoder–decoder structures. These approaches all aim to promote robust fusion between high-resolution details and low-resolution global context. However, strategies like DDMSNet and GridFormer that process multi-resolution inputs may introduce interpolation artifacts during the image scaling process, thereby interfering with or corrupting original traces of tampering. Another class of methods, such as MB-TaylorFormer V2 [26], innovates at a finer granular level by generating multi-scale representations at the token level within Transformer modules, thereby efficiently obtaining flexible receptive fields. Although these multi-scale architectures perform excellently in their respective tasks, their design philosophy is fundamentally intended to handle global or semi-global natural degradations (such as rain, snow, haze), and their inherent feature fusion logic presents a fundamental conflict with the requirements of image splicing forensics tasks. In image restoration tasks, the dominant role of multi-scale fusion is played by low-frequency global information: networks utilize stable scene structures (low-frequency) as priors to guide and constrain the reconstruction of high-frequency details, with the goal of enforcing consistency between high and low-frequency features to generate visually coherent images. However, in splicing forensics, the key evidence is precisely those high-frequency artifacts (such as splicing seams and noise anomalies) that are inconsistent with the surrounding low-frequency semantic background. Therefore, networks designed for restoration tasks, with their inherent bias toward pursuing “consistency”, tend to treat these high-frequency tampering traces as “noise” and smooth or suppress them, which runs counter to the forensic objective of preserving and amplifying “inconsistencies”. Meanwhile, strategies like DDMSNet and GridFormer that rely on input image scaling may themselves corrupt the extremely fine forensic fingerprints originating from device-level sources through their interpolation processes.

With the growing popularity of attention mechanisms and dual-branch networks, some methods add attention mechanisms to the backbone network, while others prefer to use a dual-branch backbone to extract different types of image features. Zhou et al. [19] employ a dual-stream feature synergy framework that achieves cross-modal detection and localization of tampered regions by extracting low-order noise residuals (sensor pattern noise) and high-order semantic features (deep CNN representations) in parallel and fusing them via feature concatenation. D-Net [21] adopts a dual-encoder architecture (with a fixed encoder extracting pixel structure information and a learnable encoder learning image fingerprints) and synergistically enhances the comprehensive detection capability for tampered regions through multi-level feature fusion and a spatial pyramid global feature enhancement (SPGFE) module. The ViT-VAENet model, proposed in [27], combines the self-attention mechanism of the Vision Transformer (ViT) with a Variational Autoencoder (VAE). It leverages ViT’s global context awareness and VAE’s latent feature generation capabilities to achieve localization of tampered regions in images without requiring a fixed input size. Subsequently, an increasing number of researchers have focused on methods that combine image features with edge features. MVSS-Net [5] achieves effective fusion of noise and edge features by using a Sobel filter and an additional edge-supervision branch. EMF-Net [28] better localizes tampered regions by combining RGB image features and edge features. Building on the previous two, UGEE-Net [29] adds uncertainty guidance to improve the model’s sensitivity to subtle tampering.

## 3. Methodology

To address the limitations of prior work, particularly the information isolation inherent in dual-branch architectures, we propose a novel methodological framework in this section. Unlike paradigms that process different features in separate streams, our approach is built upon a single-backbone synergistic enhancement design. The core philosophy is to create a unified process where high-level semantic understanding and low-level artifact perception are not treated as separate tasks but are deeply integrated. This is achieved through a cascade of specialized modules designed for multi-scale feature preservation, adaptive edge-aware analysis, and effective feature fusion, which will be detailed in the following subsections.

### 3.1. Overall Architecture

**Figure 2:**
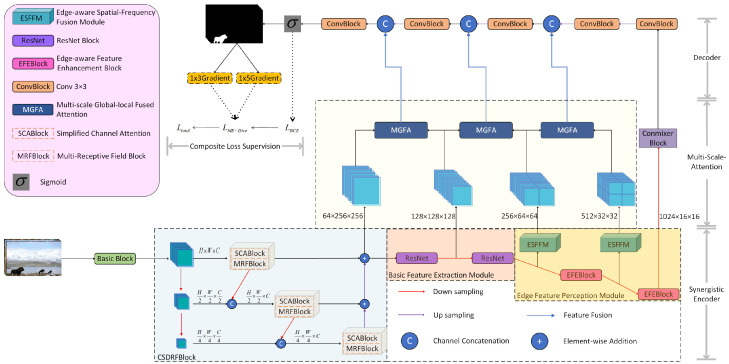
The overall architecture of the proposed AMSEANet. The network follows a standard encoder–decoder structure, incorporating three main innovative modules: the Cross-Scale Dense Residual Fusion Block (CSDRFBlock) for multi-scale feature enhancement, the Edge-Aware Spatial-Frequency Fusion Module (ESFFM) for guided trace perception, and the MGFA mechanism in the skip connections.

As shown in Figure 2, the proposed AMSEANet adopts a standard encoder–decoder architecture to efficiently learn feature distributions at various scales. The entire AMSEANet is primarily divided into three stages: semantic feature enhancement, image feature extraction, and edge feature perception. In the semantic feature enhancement stage, we use a CSDRFBlock before the first down-sampling layer to prioritize the processing of high-resolution features. This pre-emptively reinforces and preserves multi-scale contextual information before it enters the deep network, laying a solid foundation for subsequent tampering artifact detection. In the image feature extraction stage, we employ two standard residual blocks from ResNet-18 to perform deep feature extraction on the feature map output by the preceding module and to further extract image features, passing a feature map rich in semantic and image information to the next stage of edge feature perception. In the edge feature perception module, we utilize ESFFM to dynamically perceive and enhance tampering-related edge artifact information within the feature stream. Finally, skip connections are added between the down-sampling and up-sampling processes, and MGFA is used to strengthen the relationships among feature information from different scales and from different receptive fields within the same scale. This bridges the gap between high-level semantics and low-level details, ensuring the precision of the final segmentation mask.

### 3.2. Multi-Scale Global–Local Fused Attention

**Figure 3:**
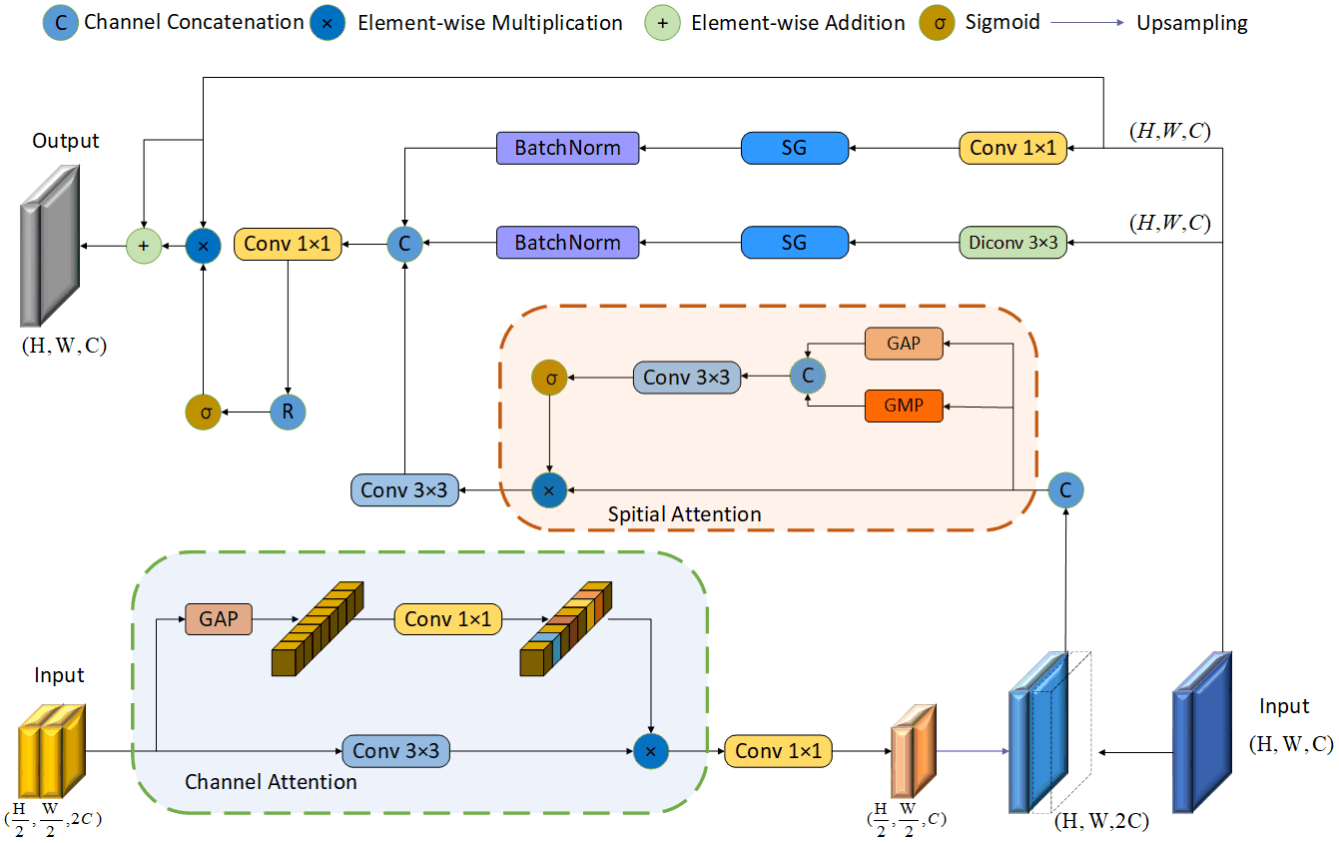
The architecture of the proposed MGFA module.

To address the issues of semantic information loss during feature extraction in the encoder architecture, weakened relationships between multi-scale features, and the lack of diversity in receptive fields at the same scale, this paper proposes a multi-scale feature enhancement attention mechanism. Its core consists of three stages that operate between the encoder and decoder: Multi-Scale Interaction, Local Receptive Field Enhancement, and Global Context Modeling. The structure is shown in Figure 3. The Multi-Scale Interaction module overcomes the limitation of traditional attention mechanisms that rely only on features from a single encoder layer by implementing cross-level feature calibration through a bi-directional channel-spatial attention mechanism to identify tampered regions. The Local Receptive Field Enhancement module is used to capture tampering trace features within the local context, while the Global Context Modeling module is responsible for extracting global features at the current level.

#### 3.2.1. Multi-Scale Feature Interaction

Given the high-level feature input $F_{\mathrm{high}}\in R^{H\;\times \;W\;\times \;C}$ from the *n*-th encoder layer and the low-level feature input $F_{\mathrm{low}}\in R^{\frac{H}{2}\;\times \;\frac{W}{2}\;\times \;2C}$ from the (n+1)-th encoder layer, a channel attention (CA) mechanism is first applied to $F_{\mathrm{low}}$. A channel attention weight vector is generated via global average pooling and a 1×1 convolution to facilitate cross-channel information selection. Subsequently, a 1×1 convolution compresses the channel dimension to *C*, and the result is upsampled to H×W resolution, yielding the refined low-level features ${\bar{F}}_{\mathrm{low}}\in R^{H\;\times \;W\;\times \;C}$. Finally, ${\bar{F}}_{\mathrm{low}}$ and $F_{\mathrm{high}}$ are concatenated along the channel dimension to obtain the fused features $F_{\mathrm{cat}}\in R^{H\;\times \;W\;\times \;2C}$, thereby achieving cross-scale feature recombination. A spatial attention (SA) mechanism is then employed to compute a pixel-wise attention map, strengthening the response in key regions. Finally, a 3×3 convolution reduces the channel dimension to *C*, outputting the multi-scale fused features $F_{\mathrm{multi}}$.

(1) $${\vec{F}}_{\mathrm{low}}={\mathrm{Conv}}_{1\times 1}\left({\mathrm{Conv}}_{1\times 1}\left(\mathrm{GAP}\left(F_{\mathrm{low}}\right)\right)⊗{\mathrm{Conv}}_{3\times 3}\left(F_{\mathrm{low}}\right)\right)$$

(2) $$F_{\mathrm{multi}}=\mathrm{sigmoid}\left({\mathrm{Conv}}_{3\times 3}\left(\left[\mathrm{GAP}\left(F_{\mathrm{cat}}\right)\right];\left[\mathrm{GMP}\left(F_{\mathrm{cat}}\right)\right]\right)\right)⊗F_{\mathrm{cat}}$$

#### 3.2.2. Local-Global Feature Focusing

To capture tampering trace features and local artifacts within the local context, a 3×3 dilated convolution with a dilation rate of 2 is applied to the original features $F_{\mathrm{high}}$. A SimpleGate gating mechanism is then used to perform feature selection and suppress irrelevant noise, outputting the locally enhanced features $F_{\mathrm{local}}$ with a channel dimension of $\frac{C}{2}$.

(3) $$F_{\mathrm{local}}=\mathrm{BatchNorm}\left(\mathrm{SimpleGate}\left({\mathrm{Conv}}_{3\times 3}^{\mathrm{dilated}}\left(F_{\mathrm{high}}\right)\right)\right)$$

Concurrently, a 1×1 convolution and a SimpleGate are employed to extract cross-channel global dependencies from $F_{\mathrm{high}}$, generating globally enhanced semantic features $F_{\mathrm{global}}$ with a channel dimension of $\frac{C}{2}$.

(4) $$F_{\mathrm{global}}=\mathrm{BatchNorm}\left(\mathrm{SimpleGate}\left({\mathrm{Conv}}_{1\times 1}\left(F_{\mathrm{high}}\right)\right)\right)$$

#### 3.2.3. Dynamic Feature Aggregation

The multi-scale features $F_{\mathrm{multi}}$, local features $F_{\mathrm{local}}$, and global features $F_{\mathrm{global}}$ are concatenated along the channel dimension. A 1×1 convolution followed by a ReLU activation function is then applied to integrate cross-dimensional information, outputting the integrated features $F_{\mathrm{fusion}}\in R^{H\;\times \;W\;\times \;C}$. Subsequently, an adaptive attention map $A\in \;{\left[0,1\right]}^{H\;\times \;W\;\times \;C}$ is generated using a Sigmoid function. The calculation process is formulated as follows:

(5) $$A=\mathrm{sigmoid}(\mathrm{ReLU}\left({\mathrm{Conv}}_{1\times 1}\left(\left[F_{\mathrm{multi}};F_{\mathrm{local}};F_{\mathrm{global}}\right]\right)\right))$$

where sigmoid(·) denotes the Sigmoid function and [;] represents the channel-wise concatenation operation. Finally, the features are enhanced via a residual connection:

(6) $$F_{\mathrm{out}}=F_{\mathrm{high}}⊗A⊕F_{\mathrm{high}}$$

where ⊗ denotes element-wise multiplication and ⊕ denotes element-wise addition. This design, through progressive weight optimization, preserves the integrity of the original features while strengthening cross-scale feature relationships and enabling comprehensive modeling of features from multiple receptive fields at the same scale.

### 3.3. Cross-Scale Dense Residual Fusion Block

Image splicing operations typically introduce visual artifacts, specifically manifested as unnatural edges and texture discontinuities in the tampered region. These artifacts provide crucial clues for the precise localization of the forgery. However, traditional convolutional neural networks (CNNs) perform poorly in the task of identifying and localizing tampered regions due to their limited receptive fields and the insufficiency of feature map information, making it difficult to effectively capture the global contextual information of the forged area. To this end, inspired by EfficientNet [30], this paper proposes an improved CSDRFBlock, which aims to enhance the network’s detection and localization accuracy for tampered regions by fusing feature information from different scales.

**Figure 4:**
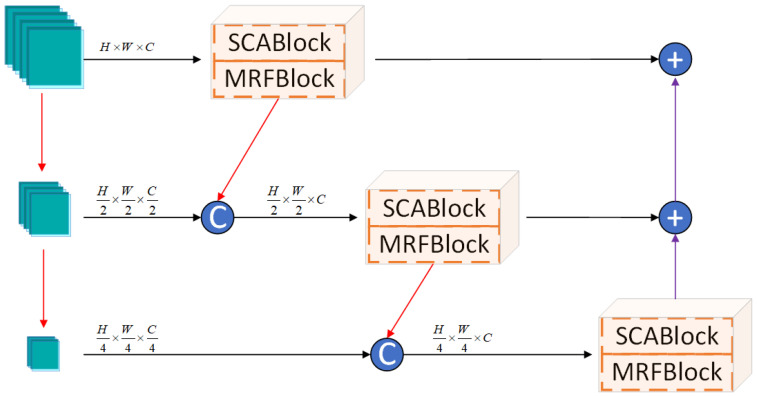
The architecture of the proposed CSDRFBlock. The blocks on the left represent the input multi-scale feature maps. The module processes them through its three main components: a multi-scale feature generation layer, a top-down cross-scale interaction branch, and a bottom-up residual enhancement path to produce the fused output. The red arrows indicate downsampling operations, while the dark red arrows within the residual path represent upsampling operations.

As shown in Figure 4, the multi-scale feature extraction module, CSDRFBlock, is primarily composed of three components: a multi-scale feature generation layer, a cross-scale interaction branch, and a bottom-up residual enhancement module. The multi-scale feature generation layer decomposes the input feature map $P\in R^{H\;\times \;W\;\times \;C}$ into a high-resolution feature map $F_{1}\in R^{H\;\times \;W\;\times \;C}$, a medium-scale feature map $F_{2}\in R^{\frac{H}{2}\;\times \;\frac{W}{2}\;\times \;\frac{C}{2}}$, and a low-scale feature map $F_{3}\in R^{\frac{H}{4}\;\times \;\frac{W}{4}\;\times \;\frac{C}{4}}$ through two progressive downsampling operations (indicated by red arrows). To ensure that each scale can maximally leverage the feature information from higher-level scales, the CSDRFBlock incorporates an inter-level feature inheritance mechanism, namely the cross-scale interaction branch. During processing in the scale space $S_{k}$ (where k=1,2,3), each scale level $S_{k}$, prior to performing feature extraction at its own scale, receives a compressed feature map $F_{k-1}^{\mathrm{down}}\in R^{H_{k}\;\times \;W_{k}\;\times \;C_{k}}$ from the higher level $S_{k-1}$ via a downsampling operation.

The process can be formulated as:

(7) $$F_{k-1}^{\mathrm{down}}=d\left(\mathrm{MRFB}\left(\mathrm{SCA}\left(F_{k-1}\right)\right)\right)$$

Here, d(·) denotes a spatial downsampling operation that includes channel compression, with the spatial dimensions defined as $H_{k}=\frac{H}{2^{k-1}}$ and $W_{k}=\frac{W}{2^{k-1}}$. This feature is then concatenated with the original input feature $F_{k}$ at the current scale along the channel dimension. This operation is represented as follows, where [;] denotes channel-wise concatenation:

(8) $$F_{k}^{\mathrm{fusion}}=\left[F_{k-1}^{\mathrm{down}};F_{k}\right]$$

To mitigate the gradient vanishing problem in deep networks, the module employs a bottom-up residual aggregation path. The output of each layer is upsampled (denoted by up(·)) and then added element-wise to the output of the preceding higher-level layer, ultimately achieving multi-dimensional enhancement of the required features. For the *i*-th branch (where i∈{2,3}), its output features are upsampled to be fused with the features from the preceding higher-level scale. The upsampling process for each branch is defined as:

(9) $$F_{2}^{\mathrm{up}}=up\left(\mathrm{MRFB}\left(\mathrm{SCA}\left(F_{2}^{\mathrm{fusion}}\right)\right)⊕F_{3}^{\mathrm{up}}\right)\in R^{H_{1}\;\times \;W_{1}\;\times \;C_{1}}$$

(10) $$F_{3}^{\mathrm{up}}=up\left(\mathrm{MRFB}\left(\mathrm{SCA}\left(F_{3}^{\mathrm{fusion}}\right)\right)\right)\in R^{H_{2}\;\times \;W_{2}\;\times \;C_{2}}$$

Finally, the features from each scale are aggregated through element-wise addition to produce the final output, *x*:

(11) $$x=\mathrm{MRFB}\left(\mathrm{SCA}\left(F_{1}\right)\right)⊕up\left(\mathrm{MRFB}\left(\mathrm{SCA}\left(F_{2}^{\mathrm{fusion}}\right)\right)⊕up\left(\mathrm{MRFB}\left(\mathrm{SCA}\left(F_{3}^{\mathrm{fusion}}\right)\right)\right)\right)$$

#### 3.3.1. SimpleGate

**Figure 5:**
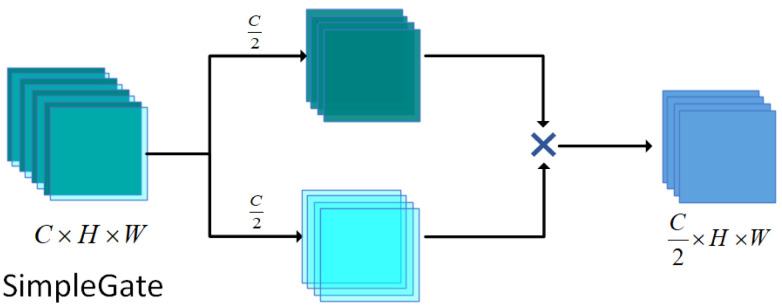
A diagram of the structure of SimpleGate.

SimpleGate is a lightweight yet efficient non-linear feature transformation module. Its core idea is to achieve adaptive feature modulation through channel splitting and element-wise multiplication (Hadamard product). As illustrated in Figure 5, given an input feature tensor $x\in R^{B\;\times \;C\;\times \;H\;\times \;W}$, where *B* represents the batch size, *C* is the number of channels, and *H* and *W* represent the spatial height and width, respectively, *x* is first split into two equal sub-tensors along the channel dimension: $x_{1},x_{2}=\mathrm{split}\left(x,\dim=1,\mathrm{parts}=2\right)$.

The gating modulation operation achieves feature interaction through the Hadamard product (⊗), and the final output SG(x) is given by:

(12) $$\mathrm{SG}\left(x\right)=x_{1}⊗x_{2}\in R^{B\;\times \;\frac{C}{2}\;\times \;H\;\times \;W}$$

By splitting the input feature map evenly into two parts, $x_{1}$ and $x_{2}$, along the channel dimension, SimpleGate forces the network to decouple two independent sets of semantic representations during the feature propagation process. This splitting essentially divides the original feature space into two complementary subspaces. The subsequent element-wise multiplication (Hadamard product) is not merely an act of information compression but rather constructs a non-linear cross-subspace interaction mechanism. The multiplication of $x_{1}$ and $x_{2}$ is equivalent to establishing an adaptive feature-selection gate along the channel dimension, enabling the network to automatically identify key response areas within both feature sets that are sensitive to the current task. Unlike traditional activation functions (such as ReLU), which perform fixed-threshold truncation, SimpleGate achieves a dynamic non-linear transformation through the multiplication of feature subspaces.

#### 3.3.2. Simplified Channel Attention Block

**Figure 6:**
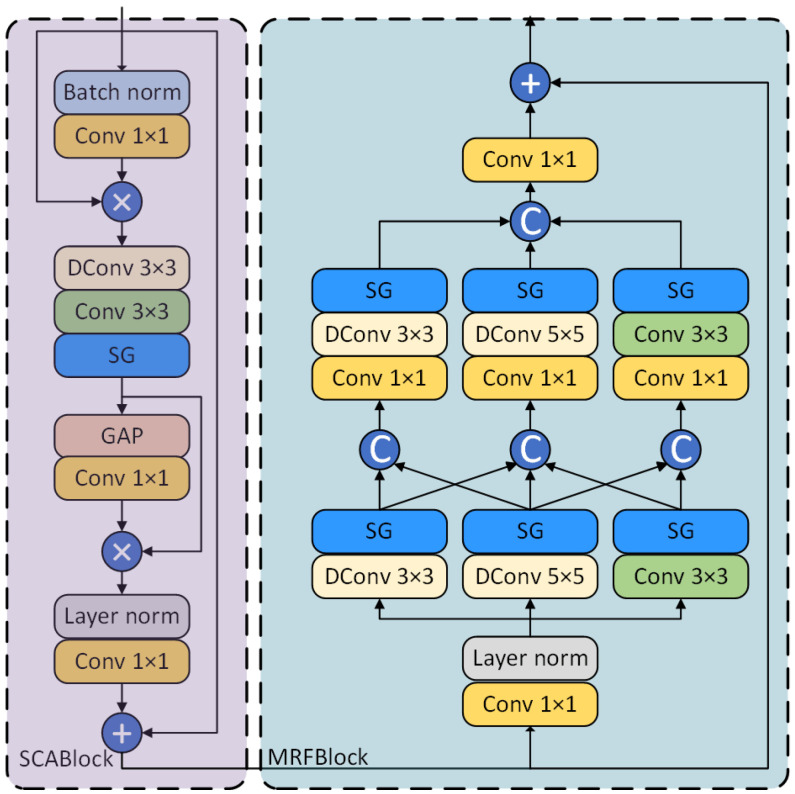
The architecture of the proposed Simplified Channel Attention Block (SCABlock) and the Multi-Receptive Field Block (MRFBlock).

As illustrated in Figure 6, the SCABlock introduces a lightweight channel attention mechanism designed to generate channel weights through global average pooling (GAP) and a series of convolutional operations, thereby effectively extracting salient features. Specifically, to stabilize the feature distribution and accelerate training, the module first normalizes the input feature map across the batch dimension using BatchNorm and then uses a 1×1 convolution to extract high-level features. Subsequently, a depth-wise convolution (DConv) and a standard convolution are employed to further extract spatial features, which are then activated through a SimpleGate (SG) module. Next, global information is acquired via global average pooling, and channel weights are subsequently adjusted using a 1×1 convolution followed by Layer Normalization, which performs normalization across the channel dimension. These weights are then multiplied by the original feature map to achieve dynamic channel-wise weighting. This design not only effectively captures the interdependencies between different channels but also enhances the feature representation capability while maintaining a lightweight model architecture.

The SCABlock ensures that key features are enhanced and redundant information is suppressed through multiple stages of feature calibration. Specifically, this includes an initial feature extraction stage, where features are preliminarily refined using BatchNorm and a 1×1 convolution; a deep feature enhancement stage, which utilizes a 3×3 depth-wise convolution and a 3×3 standard convolution to further enhance feature representation; a global information fusion stage, where GAP and a subsequent 1×1 convolution integrate global information to generate a channel attention weight vector; and a final feature calibration stage, where Layer Norm and a 1×1 convolution perform the final calibration to obtain a precise feature vector, ensuring that the output features have a stronger representative capacity. Given an input feature tensor $x\in R^{B\;\times \;C\;\times \;H\;\times \;W}$, the feature outputs $f_{i}$ from the four stages (where i∈{1,2,3,4}) can be, respectively, represented as:

(13) $$f_{1}=\mathrm{BatchNorm}\left({\mathrm{Conv}}_{1\times 1}\left(x\right)\right)⊗x$$

(14) $$f_{2}=\mathrm{SG}\left({\mathrm{Conv}}_{3\times 3}\left({\mathrm{DConv}}_{3\times 3}\left(f_{1}\right)\right)\right)$$

(15) $$f_{3}={\mathrm{Conv}}_{1\times 1}\left(\mathrm{GAP}\left(f_{2}\right)\right)⊗f_{2}$$

(16) $$f_{4}={\mathrm{Conv}}_{1\times 1}\left(\mathrm{LN}\left(f_{3}\right)\right)⊕x$$

#### 3.3.3. Multi-Receptive Field Block

As illustrated in Figure 6, the Multi-Receptive Field Block (MRFBlock) effectively captures both local and global image information by designing multiple parallel branches to process features at different scales. Specifically, the MRFBlock contains three main branches, which, respectively, employ two consecutive layers of 3×3 and 5×5 depth-wise convolutions (DConv), and a 3×3 standard convolution, combined with 1×1 convolutions and the SimpleGate (SG) activation function, to extract features from different receptive fields. After their respective convolutional operations, the branches exchange information via a cross-connection mechanism, which enhances the diversity and complementarity of the features. To ensure the thorough fusion of features from different scales, the MRFBlock is designed with an efficient internal information interaction mechanism. This includes cross-connections between the branches and the fusion of branch features using a 1×1 convolution and Layer Normalization to generate the final multi-scale feature map. Additionally, the final output of each layer within the MRFBlock is aggregated through bottom-up element-wise addition, ultimately achieving effective feature enhancement. Given the output features $f_{c}$ from the SCABlock, they are passed through Layer Normalization and a point-wise convolution before being fed into three parallel branches to obtain contextually rich features $f_{t}$. The entire process can be represented as follows:

(17) $$x^{f}=\mathrm{SG}\left({\mathrm{DConv}}_{3\times 3}\left(\mathrm{LN}\left({\mathrm{Conv}}_{1\times 1}\left(f_{c}\right)\right)\right)\right)$$

(18) $$x^{s}=\mathrm{SG}\left({\mathrm{DConv}}_{5\times 5}\left(\mathrm{LN}\left({\mathrm{Conv}}_{1\times 1}\left(f_{c}\right)\right)\right)\right)$$

(19) $$x^{t}=\mathrm{SG}\left({\mathrm{Conv}}_{3\times 3}\left(\mathrm{LN}\left({\mathrm{Conv}}_{1\times 1}\left(f_{c}\right)\right)\right)\right)$$

(20) $$\begin{array}{cc} f_{t}^{\prime }= & [\mathrm{SG}({\mathrm{DConv}}_{3\times 3}\left({\mathrm{Conv}}_{1\times 1}\left(x^{f},x^{s}\right)\right)),\\ \; & \;\mathrm{SG}({\mathrm{DConv}}_{5\times 5}\left({\mathrm{Conv}}_{1\times 1}\left(x^{f},x^{s},x^{t}\right)\right)),\\ \; & \;\mathrm{SG}({\mathrm{Conv}}_{3\times 3}\left({\mathrm{Conv}}_{1\times 1}\left(x^{t},x^{s}\right)\right))] \end{array}$$

(21) $$f_{t}={\mathrm{Conv}}_{1\times 1}\left(f_{t}^{\prime }\right)⊕f_{c}$$

This bottom-up feature aggregation strategy not only enhances the model’s performance on the image splicing detection task but also ensures the efficiency and robustness of the feature extraction process

### 3.4. Edge Feature Perception Module

**Figure 7:**
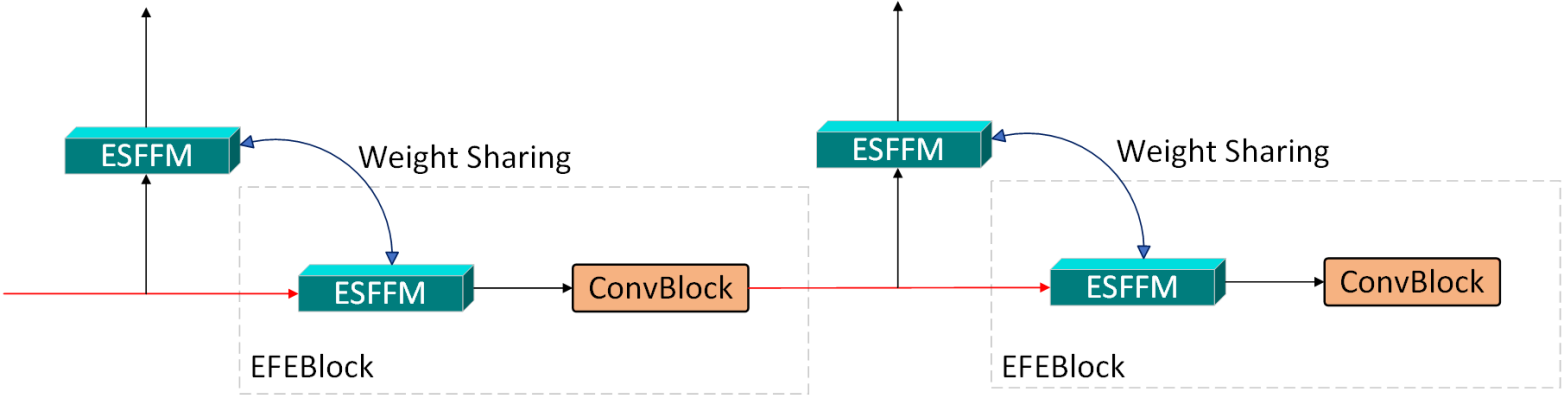
The architecture of the Edge-Aware Feature Enhancement Block (EFEBlock). The block is composed of two ESFFM modules and a ConvBlock in series, with a weight-sharing strategy applied between corresponding modules.

This module is constructed from two series-connected Edge-Aware Feature Enhancement Blocks (EFEBlocks), detailed in Figure 7, where each block comprises a core ESFFM module and a ConvBlock. The design of the core ESFFM is elaborated below.

#### 3.4.1. Edge-Aware Spatial-Frequency Fusion Module

In the image tamper detection task, the boundary between the tampered and authentic regions often contains critical traces of forgery. These traces can manifest as unnatural edge transitions, anomalous noise patterns, or subtle textural inconsistencies, and they are typically rich in frequency-domain information. For instance, EMF-Net [28] employs the Spatial Rich Model (SRM) [31], a classic forensic method that uses a set of handcrafted high-pass filters to extract noise-like features, to capture high-frequency information. However, the weights of SRM are predefined and cannot be learned or updated. To overcome this issue, MSNP-Net [18] employs a learnable constrained convolution [32] to extract high-frequency features; however, during the learning process, it tends to over-rely on high-frequency features while neglecting low-frequency ones. The research by UGEE-Net [29] demonstrated that low-frequency information, which typically includes pixels with slight intensity variations, is equally important for image splicing localization. The subtle pixel intensity changes in smooth regions, contained within low-frequency information, are crucial for a complete understanding of the image content, identifying semantic inconsistencies, and localizing tampered regions that have undergone smoothing operations.

**Figure 8:**
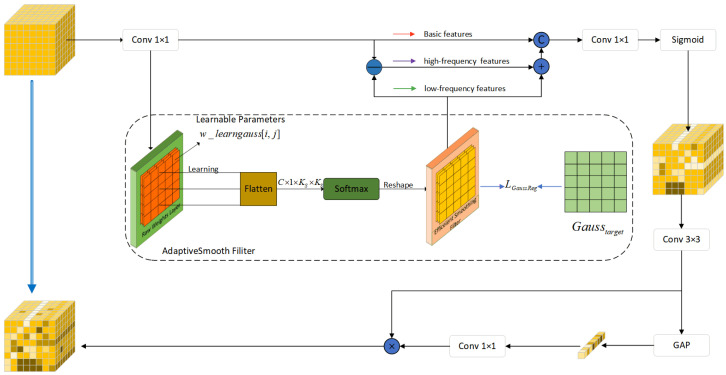
Detailed architecture of the Edge-Aware Module (ESFFM) and its core component, the Adaptive Smooth Filter (ASF). The diagram illustrates how ESFFM uses frequency decomposition to generate an attention map for feature enhancement. The dashed box details the working principle of its core component, the ASF, which dynamically generates a smoothing kernel from learnable parameters.

To effectively capture and enhance these indicative edge features, and to fully leverage information from different frequency domains, we have designed the ESFFM. As illustrated in Figure 8, the core of this module lies in the introduction of a learnable ASF, which dynamically decomposes the input features into their low-frequency (smooth) and high-frequency (detail) components based on the data. Subsequently, these separated components are combined with the original feature information and refined through subsequent convolutional and attention mechanisms, thereby achieving a comprehensive enhancement of the indicative edge tampering features.

#### 3.4.2. Adaptive Smooth Filter

The ASF aims to learn an optimal low-pass filtering operation in a data-driven manner. This unit contains a set of learnable raw weight parameters $\theta_{raw}\in R^{C\;\times \;1\;\times \;K_{s}\;\times \;K_{s}}$, where $K_{s}$ is the spatial size of the convolutional kernel (e.g., 5×5). These parameters are transformed via a Softmax function to generate an effective smoothing (low-pass) convolutional kernel $W_{eff}\in R^{C\;\times \;1\;\times \;K_{s}\;\times \;K_{s}}$. For the raw weight kernel $\theta_{raw_{i}}$ of each channel *i*, its corresponding effective kernel weight $W_{{\mathrm{eff}}_{i}}\left[j,k\right]$ is calculated as follows:

(22) $$W_{{\mathrm{eff}}_{i}}\left[j,k\right]=\frac{\exp(\theta_{\mathrm{raw}\_{\mathrm{flat}}_{i}}\left[p\left(j,k\right)\right])}{\sum_{q=1}^{K_{s}^{2}}\exp\left(\theta_{\mathrm{raw}\_{\mathrm{flat}}_{i}}\left[q\right]\right)}$$

where $\theta_{\mathrm{raw}\_{\mathrm{flat}}_{i}}$ is the flattened form of $\theta_{{\mathrm{raw}}_{i}}$, and p(j,k) is a mapping from the 2D index (j,k) to a 1D index. This Softmax transformation ensures that $W_{{\mathrm{eff}}_{i}}$ possesses the key mathematical properties of a local weighted averaging operator: non-negativity ($W_{{\mathrm{eff}}_{i}}\left[j,k\right]\geq 0$) and normalization (i.e., the weights sum to one, $\sum_{j,k}W_{{\mathrm{eff}}_{i}}\left[j,k\right]=1$). This makes the subsequent convolution operation, $X_{\mathrm{smooth}}^{\left(i\right)}=\left(W_{{\mathrm{eff}}_{i}}*X_{\exp}^{\left(i\right)}\right)+b_{{\mathrm{raw}}_{i}}$ (where * denotes the convolution operation, $X_{\exp}\in R^{B\;\times \;C\;\times \;H\;\times \;W}$ is the feature map and $b_{{\mathrm{raw}}_{i}}$ is a learnable bias), essentially an adaptive local weighted average applied to each input channel $X_{\exp}^{\left(i\right)}$, thereby yielding its low-frequency component $X_{\mathrm{smooth}}^{\left(i\right)}$. The properties of this low-pass filtering operation, $H_{LP}$, are determined entirely by the data and the learning objective, rather than being predefined. Correspondingly, the high-frequency component $X_{hf}$ can be obtained through a difference operation:

(23) $$X_{\mathrm{hf}}=X_{\exp}-X_{\mathrm{smooth}}=\left(I-H_{LP}\right)\cdot X_{\exp}=H_{HP}\cdot X_{\exp}$$

where I is the identity operator, and $H_{HP}$ is the complementary, implicitly learned adaptive high-pass filtering operator to $H_{LP}$. To ensure the smoothing kernel learned by the Adaptive Smooth Filter is robust and possesses favorable mathematical properties, we introduce an optional $L_{2}$ regularization term. This regularization term guides the learning by minimizing the mean squared error between the learned effective smoothing kernel $W_{eff}$ and a predefined target Gaussian kernel $G_{target}$ (with a standard deviation of $\sigma_{target}$):

(24) $$L_{\mathrm{GaussReg}}={∥W_{eff}-G_{target}∥}_{F}^{2}$$

This regularization can be viewed as introducing a Gaussian prior centered at $G_{target}$ into the parameter space of $W_{eff}$. This encourages the model to favor the formation of filters with classic smoothing characteristics while optimizing the main task loss.

After obtaining the adaptive low-frequency component $X_{\mathrm{smooth}}$ and high-frequency component $X_{hf}$, the ESFFM module integrates this information through a specific fusion strategy. In the current design, we first precisely reconstruct $X_{\exp}$ through the operation $X_{\mathrm{pin}}=X_{hf}+X_{\mathrm{smooth}}$. This reconstructed feature is then concatenated with the original $X_{\exp}$ along the channel dimension, forming $X_{\mathrm{fused}}=\left[X_{\mathrm{pin}};X_{\exp}\right]$. This concatenation allows a subsequent 1×1 convolutional layer to perform a weighted combination and dimensionality reduction, enabling it to learn how to optimally utilize these frequency-analyzed features derived from $X_{\exp}$.

### 3.5. Multi-Scale Edge Dice Loss

**Figure 9:**
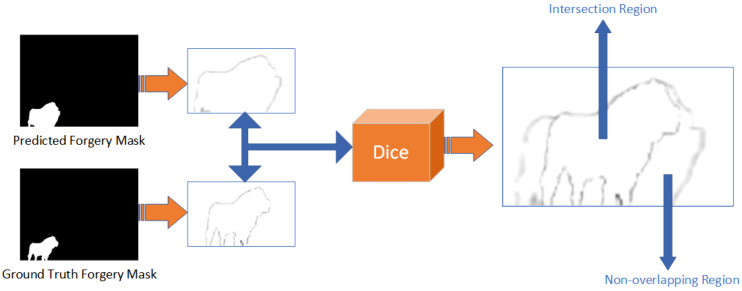
Diagram illustrating the principle of the traditional Dice loss function. The method measures the quality of a segmentation result by comparing the Predicted Forgery Mask and the Ground Truth Forgery Mask based on their intersection and non-overlapping regions.

The traditional Dice loss function [33], as illustrated in Figure 9, is primarily used to measure the overall degree of overlap between the predicted mask and the ground truth mask. A significant limitation of this method is that even suboptimal segmentation results with obvious inaccuracies can still produce a considerable overlap area. Consequently, the Dice loss value can sometimes yield misleadingly high scores, which diminishes its effectiveness as a guiding metric for model optimization, especially when dealing with cases of partially correct predictions.

Therefore, this paper proposes an improved Dice loss function, which we term the Multi-Scale Edge Dice Loss (ME-Dice). This function enhances the model’s sensitivity to key traces of forgery by applying multi-scale gradient supervision to the tampering boundaries.

In our preliminary explorations, we found that directly using the standard 2D Sobel operator to compute the edge loss yielded unsatisfactory results. We analyze that this is because the standard 2D Sobel operator structurally couples differentiation with a smoothing operation. This inherent smoothing property, while effective for suppressing noise in natural images, can conversely blur or weaken the sharp yet subtle traces of forgery that serve as key evidence in the tamper detection task. It must be emphasized that rejecting the smoothing property at the loss function level is to ensure the absolute precision of the supervisory signal. This does not contradict our strategy of using the ASF module (Section 3.4.2) to adaptively separate and utilize high- and low-frequency information during the feature extraction stage. The goal of the latter is to provide the network with rich feature representations for analysis; the roles they play and the stages at which they operate within the network are entirely different.

To address the aforementioned problem, we propose a strategy that decouples smoothing from differentiation, directly employing pure one-dimensional (1D) central difference convolutional kernels to compute the gradient, thereby maximally preserving the original sharpness of the edges. Furthermore, considering that the diversity of tampering techniques can lead to boundaries exhibiting different characteristics, we have designed two sets of 1D convolutional kernels at different scales to achieve a multi-scale perception of the tampering boundaries.

The two sets of convolutional kernels we use are defined as follows:

1. Small-scale (1×3) convolutional kernel:Used to capture the highest-frequency and sharpest pixel-level edge transitions.
  $$K_{x,1\times 3}=\left[-1,0,1\right]\;K_{y,3\times 1}={\left[-1,0,1\right]}^{T}$$
2. Large-scale (1×5) convolutional kernel: Used to perceive edges that have become relatively smooth due to post-processing, such as slight blurring or compression.
  $$K_{x,1\times 5}=\left[-1,-0.5,0,0.5,1\right]\;K_{y,5\times 1}={\left[-1,-0.5,0,0.5,1\right]}^{T}$$

Let *I* be the input image, *P* be the prediction, and *T* be the target (ground truth). The edge detection process involves convolving the image with horizontal ($K_{x}$) and vertical ($K_{y}$) convolutional kernels.

$$\begin{array}{cccc} G_{x,1\times 3} & =I*K_{x,1\times 3} & G_{x,1\times 5} & =I*K_{x,1\times 5}\\ G_{y,3\times 1} & =I*K_{y,3\times 1} & G_{y,5\times 1} & =I*K_{y,5\times 1} \end{array}$$

Next, the gradient magnitude is obtained by:

(25) $$\left\{\begin{array}{c} E_{1\times 3}\left(I\right)=\sqrt{G_{x,1\times 3}^{2}+G_{y,3\times 1}^{2}+\varepsilon }\\ E_{1\times 5}\left(I\right)=\sqrt{G_{x,1\times 5}^{2}+G_{y,5\times 1}^{2}+\varepsilon } \end{array}\right.$$

$P_{\mathrm{edge}\_1\times 3}=E_{1\times 3}\left(P\right)$ and $T_{\mathrm{edge}\_1\times 3}=E_{1\times 3}\left(T\right)$ are the predicted and target edge maps obtained using the 1×3 convolutional kernel, respectively. The same logic applies to the 1×5 convolutional kernel.

(26) $$L_{\mathrm{edge}\_1\times 3}\left(P_{\mathrm{edge}\_1\times 3},T_{\mathrm{edge}\_1\times 3}\right)=1-\frac{2\sum (P_{\mathrm{edge}\_1\times 3}\cdot T_{\mathrm{edge}\_1\times 3})+\varepsilon }{\sum P_{\mathrm{edge}\_1\times 3}+\sum T_{\mathrm{edge}\_1\times 3}+\varepsilon }$$

(27) $$L_{\mathrm{edge}\_1\times 5}\left(P_{\mathrm{edge}\_1\times 5},T_{\mathrm{edge}\_1\times 5}\right)=1-\frac{2\sum (P_{\mathrm{edge}\_1\times 5}\cdot T_{\mathrm{edge}\_1\times 5})+\varepsilon }{\sum P_{\mathrm{edge}\_1\times 5}+\sum T_{\mathrm{edge}\_1\times 5}+\varepsilon }$$

Finally, we introduce a weighting factor α to balance the two edge losses from different scales. In our experiments, we empirically set α to 0.5, aiming to assign equal importance to both the fine, high-frequency edge features (captured by the 1×3 kernel) and the smoother, wider-span edge features (captured by the 1×5 kernel). The total edge loss function, $L_{\mathrm{ME}-\mathrm{Dice}}$, is calculated as follows:

(28) $$L_{\mathrm{ME}-\mathrm{Dice}}=0.5\cdot L_{\mathrm{edge}\_1\times 3}+0.5\cdot L_{\mathrm{edge}\_1\times 5}$$

## 4. Experiments and Results Analysis

To evaluate the performance of the proposed AMSEANet, we compared it with several other image splicing forgery detection methods. We conducted a series of comparative experiments to benchmark its overall performance and ablation studies to demonstrate the effectiveness of its main components. Furthermore, robustness experiments were carried out to verify the stability of our network against various common attacks.

### 4.1. Datasets

The proposed image splicing forgery detection method is analyzed and evaluated on four public datasets: CASIA v1.0, CASIA v2.0 [34], NIST’16 [35], and COLUMBIA [36]. To prevent result bias caused by source image overlap, we adhere to a strict source-based partitioning principle for all datasets. Specifically, we ensure that all tampered samples derived from the same original authentic image are assigned exclusively to a single data split (i.e., training, validation, or test set). This strategy is designed to prevent the model from learning source-specific, non-tampering features, thereby allowing for a fairer evaluation of its generalization capability in detecting common tampering artifacts. In the COLUMBIA dataset, the spliced regions are large and simple, resulting in low detection difficulty. Compared to CASIA v1.0, CASIA v2.0 contains many more subtle forgeries. The forged images in the NIST’16 dataset have been post-processed to conceal any visible tampering traces. To ensure a comprehensive and targeted evaluation, the experiments in this paper employ two different setups. The first setup is used for the main performance evaluation, covering the “Comparison with State-of-the-Art” in Section 4.4, the “Ablation Study” in Section 4.5, and the “Effectiveness Validation of the Multi-scale Edge Dice Loss” in Section 4.6. These experiments are conducted on the CASIA v2.0, NIST’16, and COLUMBIA datasets, and data augmentation techniques such as random flipping, scaling, and rotation are applied during the training process. The second setup is used for the performance validation of a specific module, namely the “Performance Comparison of Different Frequency-Domain Information Fusion Strategies” in Section 4.7. To ensure a pure evaluation of the module’s performance, this experiment uses the CASIA v1.0 and COLUMBIA datasets with a 65% training and 35% testing split, and no data augmentation strategies are employed in this phase. Table 1 summarizes the detailed dataset partitioning for the first main experimental setup and also lists the specific attack parameters used in the subsequent “Robustness Tests” in Section 4.8.

### 4.2. Evaluation Metrics

To quantitatively evaluate the performance of our proposed AMSEANet model and other comparative methods on the task of image splicing forgery detection, we treat this task as a pixel-level binary classification problem (i.e., classifying each pixel as either “tampered” or “authentic”). We employ a series of widely recognized evaluation metrics, including Precision, Recall, and the F-measure. These metrics are calculated based on the following three fundamental quantities: the number of tampered pixels correctly detected as tampered (True Positives, TPs); the number of authentic pixels incorrectly detected as tampered (False Positives, FPs); and the number of tampered pixels incorrectly identified as authentic (false negatives, FNs).

(29) $$\begin{array}{cc} \mathrm{Precision} & =\frac{\mathrm{TP}}{\mathrm{TP}+\mathrm{FP}} \end{array}$$

(30) $$\begin{array}{cc} \mathrm{Recall} & =\frac{\mathrm{TP}}{\mathrm{TP}+\mathrm{FN}} \end{array}$$

(31) $$\begin{array}{cc} F\text{-}\mathrm{measure} & =\frac{2\times \mathrm{Precision}\times \mathrm{Recall}}{\mathrm{Precision}+\mathrm{Recall}} \end{array}$$

### 4.3. Implementation Details and Loss Function

Our model is implemented in PyTorch 2.4.1 and trained on a GeForce GTX 5070Ti GPU. To validate the model’s learning capability, we trained it from scratch without using any pre-trained weights. We use the Adam optimization algorithm to optimize the model, with an initial learning rate of 0.0002, beta parameters set to (0.5, 0.999), and a batch size of 8. The model is trained for a total of 150 epochs. A cosine learning rate scheduler is employed, and all input images are resized to 256×256 pixels. Notably, the choice of the initial learning rate is critical for the model’s final performance. Our proposed ME-Dice edge loss significantly accelerates model convergence by providing fine-grained boundary supervision. It was observed experimentally that an excessively low learning rate could cause the model to converge prematurely to a local optimum without the ability to escape. Therefore, a moderate learning rate of 0.0002 was chosen to ensure that the model, while converging rapidly, retains sufficient exploratory momentum to avoid suboptimal solutions, thereby finding a more optimal parameter space during training.

To effectively train the proposed AMSEANet model, we designed a composite loss function, $L_{\mathrm{total}}$, which is a weighted combination of the binary cross-entropy loss ($L_{BCE}$), our proposed Multi-Scale Edge Dice Loss ($L_{\mathrm{ME}-\mathrm{Dice}}$), and the $L_{2}$ regularization loss ($L_{\mathrm{GaussReg}}$) from the Adaptive Smooth Filter (ASF). The total loss function is defined as follows:

(32) $$L_{\mathrm{total}}=\lambda_{1}L_{BCE}+\lambda_{2}L_{\mathrm{ME}-\mathrm{Dice}}+\lambda_{3}L_{\mathrm{GaussReg}}$$

where $\lambda_{1}=2$, $\lambda_{2}=1$, and $\lambda_{3}=100$. Since $L_{\mathrm{GaussReg}}$ itself is relatively small, the corresponding weight $\lambda_{3}$ is set to a larger value; however, its impact on the overall loss function is negligible.

### 4.4. Comparison with State-of-the-Art Methods

To evaluate the performance of the proposed AMSEANet, we selected two traditional methods and five advanced deep learning methods as comparison algorithms: NOI [37], CFA [38], ManTra-Net [14], RRU-Net [13], C2R-Net [11], CAT-Net [39], and PSCC-Net [40].

As shown in Table 2, traditional detection methods such as NOI achieve high Recall but suffer from low Precision and F-measure. This is typically attributed to the misclassification of numerous irrelevant regions as tampered, as an excessively high Recall score loses its significance without a correspondingly high Precision. Among the deep learning-based methods, CAT-Net shows a balanced performance, notably achieving the highest Precision (0.924) on the NIST’16 dataset. This suggests the model’s strength in suppressing false positives, though its slightly lower Recall indicates it may miss some traces of tampering.

AMSEANet demonstrates competitive and comprehensive performance in these comparative experiments. On all three datasets, our model achieves the highest F-measure while maintaining a good balance between Precision and Recall. Our method achieves optimal performance on the COLUMBIA dataset, which can be attributed to AMSEANet’s simultaneous capabilities for deep semantic understanding and edge detection, enabling it to easily identify large tampered regions. On the CASIA v2.0 dataset, AMSEANet obtains the highest Precision (0.887) and F-measure (0.877). It is noteworthy that our model’s Recall (0.865) is marginally lower than that of CAT-Net (0.869); however, our Precision is substantially higher. We believe this highlights a performance trade-off on this specific dataset: CAT-Net’s higher Recall may come at the cost of lower precision, reflected in its relatively low Precision of 0.833 on this dataset, which can lead to more false positives. In contrast, AMSEANet, while maintaining a high Recall, demonstrates a stronger ability to distinguish between subtle artifacts and complex authentic textures. This gives it an advantage in providing high-confidence predictions and reducing misclassifications, ultimately leading to the best overall performance in the F-measure.

**Figure 10:**
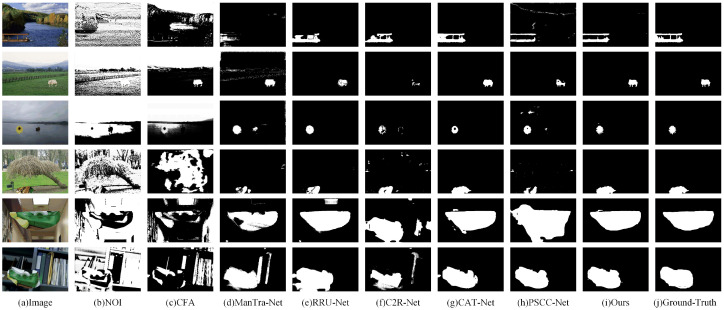
Tamper localization results of AMSEANet and other methods on public datasets. The rows, from **top** to **bottom**, show two representative samples each from the CASIA v2.0, NIST’16, and COLUMBIA datasets, respectively.

The robustness of AMSEANet is thoroughly validated on the NIST’16 dataset. Images in this dataset typically undergo complex post-processing designed to conceal overt traces of tampering, posing a significant challenge to models that rely on local edge or low-level artifacts. Consequently, we observe a notable performance degradation for some models, such as RRU-Net, whose F-measure drops from 0.843 on CASIA v2.0 to 0.765 on NIST’16. This suggests that the detection capabilities of these models are substantially compromised when low-level tampering features are suppressed. In contrast, AMSEANet demonstrates exceptional stability in this adversarial context, achieving an F-measure of 0.919 and a Recall of 0.921, both of which are the highest among all compared methods. This performance gap is attributed to our model’s advanced architecture. The effectiveness of our key modules, validated in the ablation study in Section 4.5, directly translates to this robust performance. When low-level edge cues become ambiguous or unreliable, AMSEANet can more effectively integrate high-level semantic context. For instance, the CSDRFBlock provides a high-fidelity feature foundation for the model; our ablation study has already demonstrated it to be the most critical component, improving the F-measure from 0.787 to 0.834 on its own (see Table 3). Building upon this, the ESFFM and MGFA modules further refine these features, culminating in the model’s superior ability to analyze the logical and structural plausibility of image content from a global perspective. Therefore, even when traces of tampering are intentionally concealed, our method can still effectively identify and localize the majority of forged regions, demonstrating its application potential in complex scenarios. To provide a more intuitive comparison of the localization performance of the various methods, we visualize the results on several representative samples in Figure 10.

### 4.5. Ablation Study

To systematically validate the incremental contributions of each core component in our proposed AMSEANet, we designed a series of ablation studies and comparative experiments. These experiments were conducted to verify the effectiveness of the CSDRFBlock, the ESFFM, the MGFA mechanism, and the ME-Dice. All experiments were performed on the challenging CASIA v2.0 dataset using the same training configuration to ensure a fair comparison. The detailed experimental results are presented in Table 3.

We first establish a baseline model (Baseline), a standard encoder–decoder network without any of our proposed modules, which achieves an F-measure of 0.709. By solely incorporating our proposed ME-Dice loss for edge supervision (the Edgebase model), the F-measure significantly improves to 0.787. This provides strong evidence that applying explicit supervision to tampering boundaries effectively guides the model to focus on critical artifacts, thereby substantially reducing false positives. Building upon the Edgebase, we further evaluated the individual contributions of the core modules. The introduction of CSDRFBlock yielded the largest performance gain, increasing the F-measure to 0.834. This indicates that CSDRFBlock, through its efficient cross-scale feature fusion and residual connection mechanisms, builds more robust multi-level feature maps, significantly enhancing the network’s ability to perceive and capture tampered regions of various sizes. When the ESFFM module was introduced (F-measure of 0.811), the performance improvement was primarily attributed to the spatial-frequency decomposition performed by its integrated ASF, which enables the network to accurately isolate and focus on high-frequency edge artifacts indicative of tampering. This study also reveals the synergistic effects between modules, particularly the interaction between CSDRFBlock and MGFA. By comparing the Edgebase + MGFA + ESFFM model (F-measure of 0.839) with our full model (F-measure of 0.877), it is evident that adding CSDRFBlock to the former configuration provides a substantial performance boost. This clearly demonstrates that CSDRFBlock serves as a foundational module providing high-fidelity feature streams for the subsequent MGFA. Building on this foundation, MGFA can then effectively strengthen the correlations between features from different scales and receptive fields to ensure optimal fusion of high-resolution boundary details, with both modules working in concert to unleash their full potential. These results ultimately validate our design rationale: each of our proposed modules is individually effective, and the synergy between them is key to the model’s final leading performance.

### 4.6. Effectiveness Validation of the Multi-Scale Edge Dice Loss

To systematically validate the effectiveness of our proposed ME-Dice and demonstrate its superiority over traditional loss functions, we designed the following ablation study. All experiments were conducted on the CASIA v2.0 dataset, with the baseline model uniformly using the Binary Cross-Entropy (BCE) loss as the base loss.

The experimental results in Table 4 clearly reveal the contribution of each component of our proposed loss function. First, compared to the baseline model that only uses $L_{BCE}$, the addition of any edge loss improves the model’s performance. Notably, both of our proposed single-scale 1D gradient losses (with F-scores of 0.859 and 0.865, respectively) significantly outperform the edge loss using the standard 2D Sobel operator (F-score of 0.831). This strongly validates our core hypothesis: that decoupling the smoothing and differentiation operations can more effectively supervise the network in learning tampering artifacts.

It is worth noting that the two single-scale gradient kernels exhibit distinct complementary characteristics. The 1×5 scale loss, $L_{\mathrm{edge}\_1\times 5}$, achieves the highest Precision (0.924), indicating that its wider receptive field and smoother gradient calculation enable it to robustly identify tampering boundaries and avoid misclassifying noise as artifacts. Meanwhile, the 1×3 scale loss, $L_{\mathrm{edge}\_1\times 3}$, obtains the highest Recall (0.876), demonstrating that its sensitivity to high-frequency, sharp changes allows it to maximally detect all potential tampered regions.

**Figure 11:**
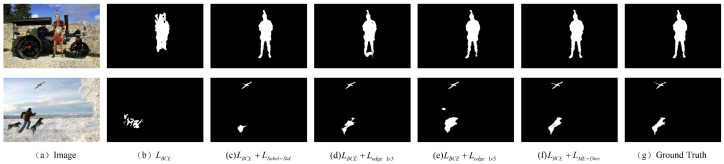
Visual comparison of the loss function ablation study. (**a**) Input image and (**g**) ground truth. (**b**–**f**) show the prediction results under different loss function configurations, where (**f**) is the result from our final Multi-Scale Edge Dice loss (ME-Dice).

Ultimately, our proposed Multi-Scale Edge Dice Loss, $L_{\mathrm{ME}-\mathrm{Dice}}$, achieves the highest F-measure (0.877), outperforming any single component. This clearly demonstrates the synergistic effect of the multi-scale strategy. By combining the high Precision of the 1×5 kernel with the high Recall of the 1×3 kernel, $L_{\mathrm{ME}-\mathrm{Dice}}$ strikes the optimal balance between precision (“getting it right”) and completeness (“finding it all”), thereby achieving the best overall tamper localization performance. Figure 11 visually demonstrates this progressive performance improvement, where the result from our final scheme most closely approximates the ground truth mask in terms of boundary details.

To more comprehensively evaluate the performance of the ME-Dice loss, we further compared it against three advanced loss functions widely used in related fields: Boundary Loss, a classic boundary-refinement loss; Focal Loss, a popular solution for addressing class imbalance; and Active Contour Loss, which is inspired by traditional contour models. As shown in Table 5, we compared these three loss functions with our final ME-Dice.

The experimental results indicate that all three advanced loss functions achieved competitive performance, with F-measures of 0.849, 0.837, and 0.859, respectively. All of them significantly outperform the traditional Sobel-based method, demonstrating their effectiveness. Among them, Active Contour Loss exhibits the highest precision (0.894), albeit with a relatively lower recall. Despite the strong performance of these methods, our ME-Dice, specifically tailored for the tampering detection task, still achieved the best overall performance with the highest F-measure of 0.877, striking a better balance between precision and recall. This comprehensively demonstrates that our strategy of using multi-scale gradient supervision to precisely capture traces of tampering is more suitable for this task compared to general-purpose boundary refinement or class-balance losses.

### 4.7. Performance Comparison of Different Frequency-Domain Information Fusion Strategies

**Figure 12:**
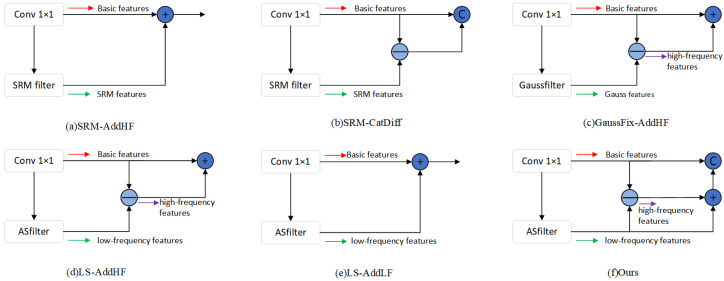
Comparative diagrams of different frequency-domain information fusion strategies. The figure illustrates the six different feature fusion methods based on fixed filters (SRM, Gaussian filter) and the adaptive filter (ASFilter) used in the comparative experiments.

To validate the synergistic effect of multi-frequency information and the role of the Adaptive Smooth Filter, as illustrated in Figure 12, we designed a series of comparative experiments. We compare our method with several alternative frequency-domain information fusion schemes on two datasets of varying difficulty (CASIA v1.0 and COLUMBIA). The CASIA v1.0 dataset contains a total of 461 images; we partitioned it, along with the COLUMBIA dataset, into a 65% training set and a 35% testing set. It should be noted that the experimental setup here is used only for the module comparison in this section and differs from the main experiments in previous sections. All comparison schemes are implemented on the same baseline network architecture, differing only in the feature fusion module. The specific descriptions of each comparison method are as follows:

Normal: As the baseline model, this method only uses basic semantic features extracted via a 1×1 convolution, without introducing any additional frequency-domain information.
SRM-AddHF: This method uses a fixed Spatial Rich Model (SRM) filter to extract high-frequency (HF) features from the image, which are then fused with the basic semantic features via element-wise addition.
SRM-CatDiff: This method first extracts high-frequency features using an SRM filter, then calculates the difference map between them and the basic semantic features. This difference map is then concatenated with the original basic semantic features along the channel dimension.
GaussFix-AddHF: This method utilizes a fixed Gaussian blur kernel to extract the low-frequency (LF) components of the input. The high-frequency features are obtained by subtracting the low-frequency components from the basic semantic features. Finally, the extracted high-frequency features are added element-wise to the basic semantic features.
LS-AddHF: This method replaces the fixed Gaussian kernel with our proposed Adaptive Smooth Filter (ASF) to learn and extract low-frequency features. Subsequently, the high-frequency features are obtained via a difference operation and added to the basic semantic features.
LS-AddLF: This method directly fuses the low-frequency features, which are adaptively learned by the ASF, with the basic semantic features via element-wise addition.
Ours: This is the final scheme proposed in this paper. First, the ASF is utilized to adaptively extract the low-frequency component, and the high-frequency component is obtained by taking the difference from the basic semantic features. Next, the low- and high-frequency components are added together to reconstruct the features. Finally, this reconstructed feature is concatenated with the original basic semantic features along the channel dimension, aiming to provide the network with a dual view that contains both original semantics and explicit frequency decomposition information.

The experimental results in Table 6 show that the baseline Normal method performs reasonably well on both datasets, demonstrating the fundamental effectiveness of deep semantic features for the tamper detection task. However, its performance also indicates that relying solely on semantic information is insufficient to capture all tampering traces accurately. Compared to the baseline, the performance of methods using fixed filters, such as SRM-AddHF and GaussFix-AddHF, degrades. This highlights a core deficiency of fixed filters: their weights are predefined and non-learnable, rendering them unable to adapt to varying image content and diverse tampering techniques. Simply superimposing fixed high-frequency details onto semantic features can introduce task-irrelevant interference, which may confuse the model and lower its precision. Notably, the SRM-CatDiff method outperforms other fixed-filter schemes. By computing the difference between basic semantic features and SRM-derived high-frequency features, it effectively generates an “inconsistency map” that explicitly highlights potential tampering traces. Although this difference-based strategy is more informative than direct addition, its ultimate effectiveness remains constrained by the non-adaptive nature of the SRM filter.

In sharp contrast, both the LS-AddHF and LS-AddLF methods, which employ our proposed Adaptive Smooth Filter (ASF), achieve significant performance improvements on both datasets. This strongly demonstrates that using a learnable, data-driven filter to dynamically separate high- and low-frequency information is a correct and efficient strategy. The model can adaptively adjust its filtering based on the input content to extract the features most relevant to the tampering task. Our proposed final scheme, designated as ‘ours’, stands out among all compared methods by achieving the best overall performance on both datasets. On the more challenging CASIA dataset, its Precision reaches 0.8671, significantly surpassing all other methods and showcasing a strong capability for suppressing false positives. On the less difficult COLUMBIA dataset, our scheme maintains its leading edge, even as the performance gap between the methods narrows.

### 4.8. Robustness Tests

To further validate the effectiveness and robustness of AMSEANet, we compared its performance against existing detection methods under various types of attacks: noise addition, JPEG compression, and resizing. It is noteworthy that none of the test sets used for the robustness experiments were included during training.

**Figure 13:**
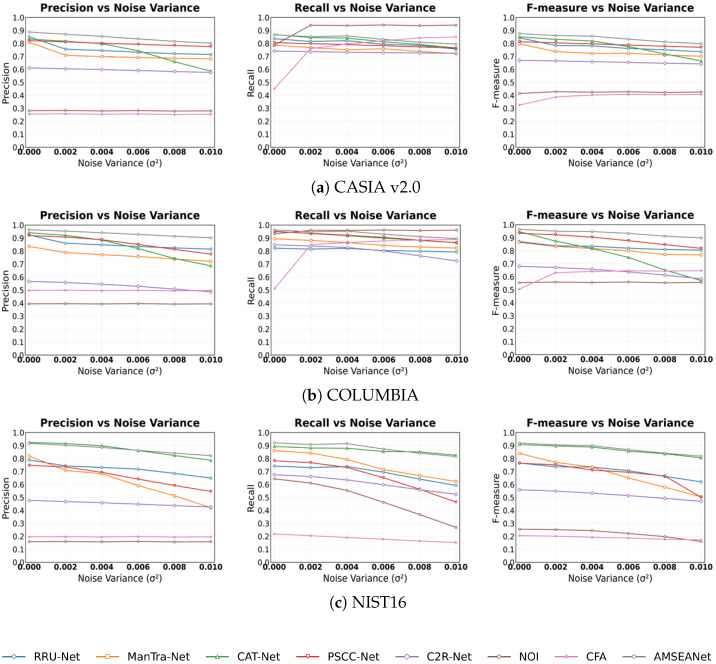
Comparison results under noise addition attack. The three columns represent Precision, Recall, and F-measure, respectively. (**a**–**c**) show the experiment results on the CASIA v2.0, COLUMBIA, and NIST16 datasets, respectively.

Noise is often introduced during image transmission or post-processing, which can interfere with a detection model’s ability to judge underlying artifacts. Therefore, a model’s stability against noise is an important performance indicator. In this experiment, we added Gaussian white noise with varying variances to the test images. As seen in the experimental results in Figure 13, the Recall of traditional methods increases dramatically, as they tend to classify nearly the entire image as tampered under severe noise attacks. Among the CNN-based methods, AMSEANet achieves the highest Precision and F-measure on both the CASIA v2.0 and COLUMBIA datasets, and its performance is comparable to that of CAT-Net on NIST’16, demonstrating its robustness against noise attacks across the three datasets.

**Figure 14:**
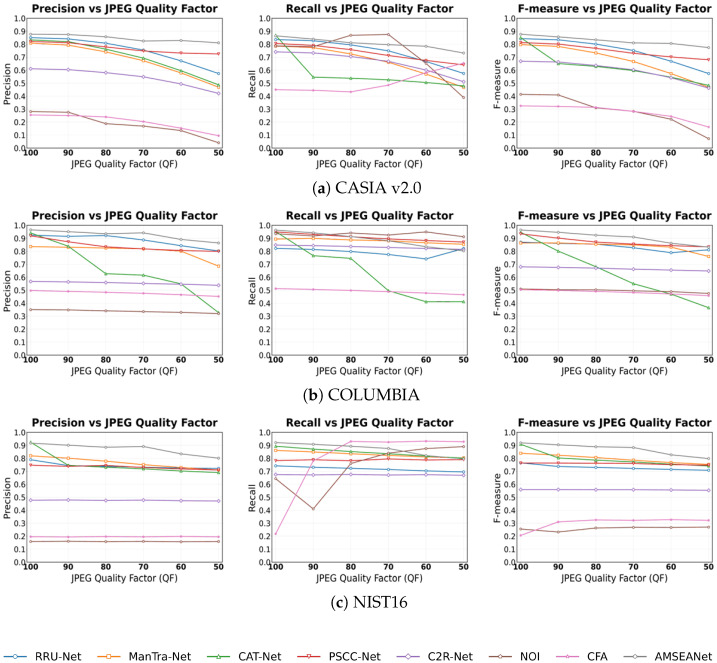
Comparison results under JPEG compression attack. The three columns represent Precision, Recall, and F-measure, respectively. (**a**–**c**) show the experiment results on the CASIA v2.0, COLUMBIA, and NIST16 datasets, respectively.

JPEG compression is a common attack used to evaluate the robustness of image tamper detection models and is ubiquitous in real-world applications. Therefore, we tested various models under different levels of JPEG compression. As shown in Figure 14, we observe that JPEG compression severely impacts the performance of all models on the CASIA v2.0 dataset, with some models exhibiting a precipitous drop in their detection rates for tampered pixels. The impact of JPEG compression is less pronounced on the COLUMBIA and NIST’16 datasets. This is because the tampered regions in COLUMBIA are large and easily discernible, while the NIST’16 dataset already consists of images that have undergone multiple post-processing steps, presenting a high level of intrinsic difficulty. Notably, the performance of CAT-Net, which is otherwise robust, degrades significantly under JPEG compression. This is likely because the JPEG attack directly disrupts the analytical basis for CAT-Net’s DCT stream by overwriting the original, fine-grained “frequency fingerprints”. In contrast, PSCC-Net constructs a dual-verification system using cross-scale relationships and Self-Correlation Calculation (SCC). By examining the consistency of image statistical features from both inter-scale and intra-scale dimensions, it can accurately decouple local, non-stationary artifacts originating from tampering from the global, stationary artifacts caused by JPEG compression, thus exhibiting more stable performance. Generally, as the Quality Factor (QF) decreases, the Precision and F-measure of most models decline. Among all the compared models, AMSEANet is less affected than its counterparts. While its Recall is slightly lower than that of the traditional methods, this is due to the high rate of false positives in the latter. Overall, AMSEANet maintains commendable robustness against JPEG compression attacks.

**Figure 15:**
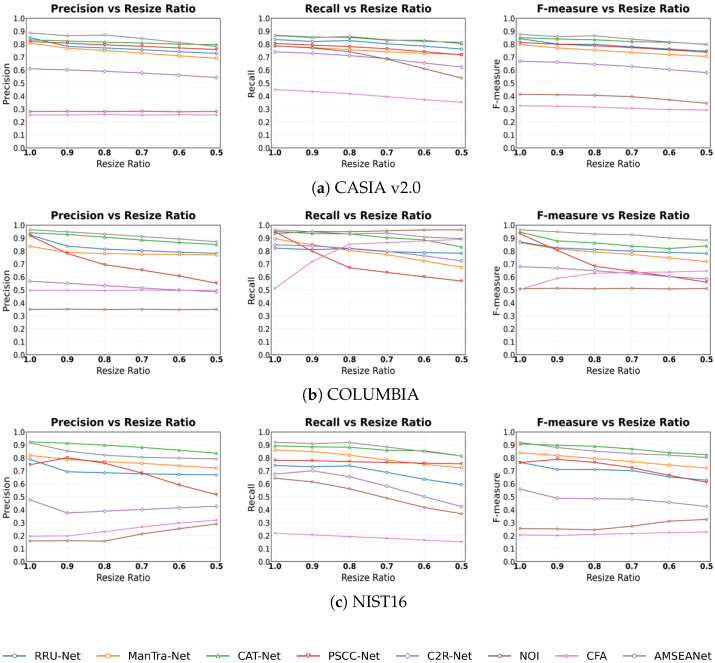
Comparison results under resize operation attack. The three columns represent Precision, Recall, and F-measure, respectively. (**a**–**c**) show the experiment results on the CASIA v2.0, COLUMBIA, and NIST16 datasets, respectively.

Image scaling, a common post-processing technique, poses a unique challenge as its inherent interpolation and resampling processes can alter crucial forensic traces. As shown in Figure 15, all deep learning-based methods exhibit some performance degradation under scaling attacks. Nevertheless, our AMSEANet demonstrates considerable stability across most scenarios, maintaining a high level of performance on the CASIA v2.0 and COLUMBIA datasets. However, on the NIST’16 dataset, our model’s performance is slightly inferior to that of CAT-Net. We attribute this phenomenon to a direct conflict between the attack’s nature and our model’s core detection mechanisms. Specifically, the interpolation process tends to blur the sharp, high-frequency artifacts found at tampering boundaries. This directly compromises the effectiveness of our Edge-Aware Spatial-Frequency Fusion Module (ESFFM), which is designed to adaptively separate these very high-frequency signals. When the original tampering artifacts are weakened, or masked by new, globally consistent interpolation artifacts, the discriminative ability of the ESFFM is inherently limited. This effect is particularly pronounced on the heavily post-processed NIST’16 dataset, which explains the model’s slightly inferior performance in this specific scenario. Despite this vulnerability in a specific context, AMSEANet’s overall strong performance across different datasets and scaling ratios validates its robust design against this challenging attack.

### 4.9. Computational Complexity Analysis

In addition to detection performance, the computational complexity of a model is a crucial indicator of its practical value. To this end, we evaluated the parameters, FLOPs, and inference speed of AMSEANet and compared them with several mainstream methods, with the results presented in Table 7. For an input size of 256 × 256, our model has 52.08 M parameters and a computational load of 124.86 GFLOPs. On a single NVIDIA GeForce RTX 5070 Ti GPU, its inference speed reaches 35.1 FPS.

The comparative analysis in Table 7 reveals the design trade-offs between performance and efficiency among different models. Compared to lightweight models such as ManTra-Net and PSCC-Net, AMSEANet has a relatively larger parameter count and computational load. We believe this moderate increase in complexity is an important factor for the model to achieve better detection performance. As the results in Table 2 show, the detection accuracy of AMSEANet, especially on challenging datasets like NIST’16, outperforms these lightweight models.

On the other hand, compared to the heavyweight model MVSS-Net (146.88 M), our AMSEANet is more compact in model size and more parameter-efficient, while its practical inference speed (35.1 FPS vs. 37.3 FPS) remains at a comparable level.

In conclusion, we believe that AMSEANet makes a reasonable trade-off between performance and complexity. While ensuring a necessary level of architectural complexity, it achieves competitive detection performance and avoids the parameter redundancy of oversized models, striking a favorable balance among accuracy, efficiency, and model size.

### 4.10. Limitations Analysis

**Figure 16:**
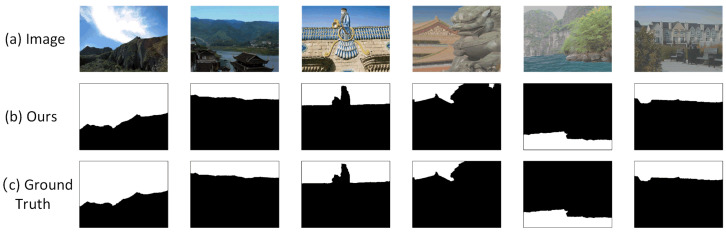
Successful detection results of AMSEANet on natural background replacement samples. Subfigure (**a**) shows the original images, while the model’s prediction (**b**) is highly consistent with the ground truth (**c**), demonstrating its effectiveness.

In our research, we noted that tampering involving large-area natural background replacements (e.g., sky, water) poses a significant challenge to current detection algorithms. Our proposed AMSEANet model makes some useful attempts to address this problem. In many test samples, the model can perform relatively accurate segmentation along the complex natural contours of foreground objects (such as mountains or buildings). As shown in Figure 16, the detection results in these cases are highly consistent with the ground truth, which preliminarily demonstrates the potential of our designed method.

**Figure 17:**
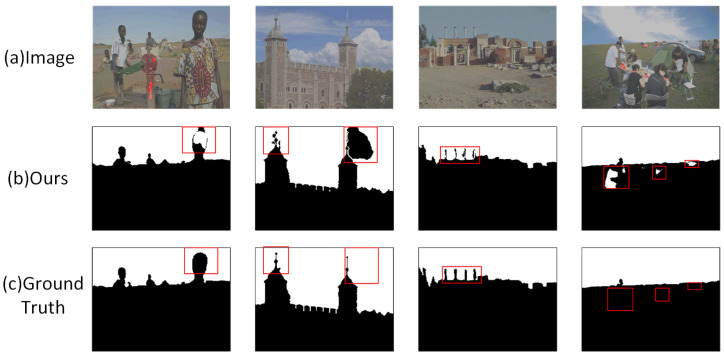
Limitation analysis of AMSEANet on highly challenging samples. Subfigure (**a**) shows the original images, while red boxes highlight discrepancies between the prediction (**b**) and the ground truth (**c**), showing that the model may produce incomplete detections on contours with extremely fine-grained details.

Of course, we must also acknowledge that the model’s performance still has room for improvement when the complexity and intricacy of these natural boundaries reach extremely high levels. We preliminarily attribute the detection difficulties in such scenarios to the following two aspects:

1. Challenges from visual realism and low-contrast boundaries: This type of splicing often achieves a high degree of realism by replacing the background, which occupies a large portion of the image. Forgers use advanced post-processing techniques that result in extremely low contrast at the boundary between the forged background and the authentic foreground, making it visually seamless. This renders detection methods based on local artifacts highly prone to failure.
2. Segmentation challenges from the highly complex contours of authentic foregrounds: The core difficulty of the task lies in the fact that the model does not need to segment a newly introduced forgery edge, but rather the natural, extremely complex, and irregular contour of the authentic foreground object itself. As shown in Figure 17, the fine structure of a castle turret or the minute contour of a distant person are authentic yet extremely complex details preserved from the original image. The algorithm must perfectly delineate these details, much like tracing an outline, to accurately define the replaced background area. This places extremely high demands on the model’s edge perception and precise localization capabilities.

As can be seen from the areas marked by red boxes in Figure 17, when faced with fine structures like the castle turret or the contour of a small, distant person, our model sometimes produces partial missed detections (false negatives) and incomplete results. This indicates that while AMSEANet can handle complex boundaries overall, there are still shortcomings in its precision for capturing fine-grained details.

In summary, although AMSEANet has made some exploratory efforts and achieved certain results in handling natural background replacement tasks, there is still room for improvement in its robustness and precision when faced with complex, authentic contours featuring extremely fine-grained details and very low contrast. This also points the way for our future research: how to enhance the model’s comprehensive perception of weak signals and its segmentation precision for ultra-fine-grained structures.

## 5. Conclusions

To address the challenges of image splicing tamper detection, this paper proposes an Adaptive Multi-Scale Edge-Aware Network (AMSEANet). This network employs a novel single-backbone synergistic enhancement framework, which discards the traditional parallel dual-branch structure and achieves a deep integration of several key technologies within a unified feature stream. Specifically, the framework first ensures the high-fidelity transmission of micro-level tampering traces within the deep network via the Cross-Scale Dense Residual Fusion Block (CSDRFBlock). Subsequently, the Edge-Aware Spatial-Frequency Fusion Module (ESFFM) utilizes its integrated data-driven Adaptive Smooth Filter (ASF) to precisely separate and focus on high- and low-frequency tampering artifacts from the enhanced features. Concurrently, in the skip connections of the decoding path, the MGFA effectively establishes multi-level feature relationships between the tampered regions and the background. Finally, the entire network is supervised end-to-end by a custom Multi-Scale Edge Dice Loss (ME-Dice) to enhance the localization precision of tampering boundaries. Comprehensive experimental evaluations on several public benchmark datasets demonstrate that the performance of AMSEANet surpasses that of various existing state-of-the-art methods, and it exhibits superior robustness against common attacks such as noise and JPEG compression. Despite its excellent performance, the model still has limitations: its performance degrades when facing image scaling attacks; concurrently, on some datasets, the model achieves high Precision at the cost of a slightly lower Recall, revealing a potential trade-off between suppressing false positives and ensuring detection completeness.

## Figures and Tables

**Table 1:** Dataset partitioning and parameter settings for robustness tests.

Sets		Param	Range	Step	CASIA V2.0	COLUMBIA	NIST’16
Training		-	-	-	1300	125	180
Validation		-	-	-	100	10	15
Testing	Plain	-	-	-	200	45	60
	JPEG compression	QF	50–90	10	200 × 5	45 × 5	60 × 5
	Noise addition	Var	0.002–0.01	0.002	200 × 5	45 × 5	60 × 5
	Resize operation	Ratio	0.5–0.9	0.1	200 × 5	45 × 5	60 × 5
All images		-	-	-	4600	855	1155

**Table 2:** Performance comparison of our proposed method (Ours) against several state-of-the-art forgery detection methods. The best results are highlighted in bold.

Method	Detection Result								
	CASIA v2			COLUMBIA			NIST’16		
	Precision	Recall	F	Precision	Recall	F	Precision	Recall	F
NOI	0.281	0.787	0.414	0.394	0.932	0.554	0.159	0.643	0.255
CFA	0.255	0.450	0.325	0.497	0.512	0.504	0.196	0.218	0.206
ManTra-Net	0.809	0.786	0.797	0.836	0.894	0.864	0.819	0.861	0.839
RRU-Net	0.851	0.836	0.843	0.924	0.822	0.871	0.789	0.742	0.765
C2R-Net	0.611	0.741	0.669	0.567	0.848	0.680	0.477	0.675	0.559
CAT-Net	0.833	**0.869**	0.851	0.941	0.952	0.946	**0.924**	0.893	0.908
PSCC-Net	0.821	0.806	0.813	0.919	0.949	0.934	0.747	0.782	0.764
Ours	**0.887**	0.865	**0.877**	**0.965**	**0.963**	**0.964**	0.917	**0.921**	**0.919**

**Table 3:** Results of the ablation study on the core components of AMSEANet, conducted on the CASIA v2.0 dataset. The checkmark (🗸) indicates that the module is included in the configuration, while the cross (×) indicates its exclusion.

Method Set	ME-Dice	CSDRF	ESFFM	MGFA	Precision	Recall	F
Baseline	×	×	×	×	0.703	0.715	0.709
Edgebase	🗸	×	×	×	0.794	0.781	0.787
Edgebase + ESFFM	🗸	×	🗸	×	0.801	0.821	0.811
Edgebase + MGFA	🗸	×	×	🗸	0.810	0.798	0.804
Edgebase + CSDRF	🗸	🗸	×	×	0.837	0.832	0.834
Edgebase + CSDRF + MGFA	🗸	🗸	×	🗸	0.859	0.845	0.852
Edgebase + CSDRF + ESFFM	🗸	🗸	🗸	×	0.869	0.858	0.863
Edgebase + MGFA + ESFFM	🗸	×	🗸	🗸	0.835	0.843	0.839
Ours	🗸	🗸	🗸	🗸	0.887	0.865	0.877

**Table 4:** Ablation study for the edge-guided loss function. $L_{\mathrm{Sobel}-\mathrm{Std}}$ denotes the edge loss using the standard 2D Sobel operator. $L_{\mathrm{edge}\_1\times 3}$ and $L_{\mathrm{edge}\_1\times 5}$ represent the edge losses using only the 1 × 3 and 1 × 5 1D gradient kernels, respectively. $L_{\mathrm{ME}-\mathrm{Dice}}$ is the combination of $L_{\mathrm{edge}\_1\times 3}$ and $L_{\mathrm{edge}\_1\times 5}$.

Loss Composition	Precision	Recall	F
$L_{BCE}$	0.881	0.720	0.792
$L_{BCE}+L_{\mathrm{Sobel}-\mathrm{Std}}$	0.917	0.759	0.831
$L_{BCE}+L_{\mathrm{edge}\_1\times 3}$	0.843	0.876	0.859
$L_{BCE}+L_{\mathrm{edge}\_1\times 5}$	0.924	0.813	0.865
$L_{BCE}+L_{\mathrm{ME}-\mathrm{Dice}}$	0.887	0.865	0.877

**Table 5:** Performance comparison of the ME-Dice loss against other advanced loss functions. The best results are highlighted in bold.

Loss Composition	Precision	Recall	F-Measure
$L_{BCE}$ + Boundary Loss	0.851	0.847	0.849
$L_{BCE}$ + Focal Loss	0.854	0.821	0.837
$L_{BCE}$ + Active Contour Loss	**0.894**	0.827	0.859
$L_{BCE}$ + $L_{ME-Dice}$	0.887	**0.865**	**0.877**

**Table 6:** Performance comparison of different frequency-domain information fusion strategies. The best results are highlighted in bold.

Method	Detection Result					
	CASIA v1			COLUMBIA		
	Precision	Recall	F	Precision	Recall	F
Normal	0.835	0.672	0.745	0.962	0.922	0.941
SRM-AddHF	0.783	0.698	0.738	0.923	0.941	0.932
SRM-CatDiff	0.828	0.695	0.755	0.964	0.922	0.942
GaussFix-AddHF	0.805	0.676	0.735	0.878	0.930	0.903
LS-AddHF	0.833	0.702	0.762	0.963	0.923	0.943
LS-AddLF	0.806	**0.713**	0.757	0.950	0.939	0.945
Ours	**0.867**	0.687	**0.767**	**0.964**	**0.942**	**0.953**

**Table 7:** Computational complexity comparison of different methods.

Method	Parameters (M)	FLOPs (G)	FPS
MVSS-Net [5]	146.88	40.89	37.3
ManTra-Net [14]	3.80	67.90	26.4
PSCC-Net [40]	2.75	105.72	40.7
AMSEANet	52.08	124.86	35.1

## Data Availability

The data are available upon request to the correspondence e-mail.

## Abbreviations

**Abbreviation**	**Full Name**
AMSEANet	Adaptive Multi-Scale Edge-Aware Network
ASF	Adaptive Smooth Filter
CSDRFBlock	Cross-Scale Dense Residual Fusion Block
ESFFM	Edge-Aware Spatial-Frequency Fusion Module
MGFA	Multi-Scale Global–local Fused Attention
ME-Dice	Multi-Scale Edge Dice Loss
CNN	Convolutional neural network
FCN	Fully Convolutional Network
SPN	Sensor pattern noise
DCT	Discrete Cosine Transform
DMWT	Discrete Multiwavelet Transform
SRM	Spatial Rich Model (Filter)
CA	Channel attention
SA	Spatial attention
GAP	Global average pooling
GMP	Global Max Pooling
TP	True Positives
FP	False Positives
FN	False Negatives
BCE	Binary Cross-Entropy (Loss)
QF	Quality Factor
